# Helix 1.0: An open-source framework for reproducible and interpretable machine learning on tabular scientific data

**DOI:** 10.1016/j.patter.2026.101536

**Published:** 2026-04-01

**Authors:** Eduardo Aguilar-Bejarano, Daniel Lea, Karthikeyan Sivakumar, Jimiama M. Mase, Reza Omidvar, Ruizhe Li, Troy Kettle, James Mitchell-White, Morgan R. Alexander, David A. Winkler, Grazziela Figueredo

**Affiliations:** 1 Digital Research Service, University of Nottingham, Kings Meadow Campus, Lenton Lane, Nottingham NG7 2NR, UK; 2 School of Computer Science, University of Nottingham, Jubilee Campus, Wollaton Road, Nottingham NG8 1BB, UK; 3 Health Informatics, School of Medicine, University of Nottingham, Medical School, Nottingham NG7 2UH, UK; 4 School of Pharmacy, University of Nottingham, University Park, Nottingham NG7 2RD, UK; 5 Department of Biochemistry and Chemistry, La Trobe Institute for Molecular Sciences, La Trobe University, Bundoora, VIC 3086, Australia; 6 Monash Institute of Pharmaceutical Sciences, Monash University, Parkville, VIC 3052, Australia

**Keywords:** data provenance, FAIR, machine learning, reproducibility, QSAR modeling, QSPR modeling, healthcare data modeling, machine learning interpretability

## Abstract

Helix is an open-source, extensible, Python-based software framework to facilitate reproducible and interpretable machine learning workflows for tabular data. It addresses the growing need for transparent experimental data analytics provenance, ensuring that the entire analytical process—including decisions around data transformation and methodological choices—is documented, accessible, reproducible, and comprehensible to relevant stakeholders. The platform comprises modules for standardized data preprocessing, visualization, machine learning model training, evaluation, interpretation, results inspection, and model prediction for unseen data. To further empower researchers without formal training in data science to derive meaningful and actionable insights, Helix features a user-friendly interface that enables the design of computational experiments and inspection of outcomes, including a novel interpretation approach to machine learning decisions using linguistic terms all within an integrated environment.

## Highlights

• Modular, extensible architecture for data analysis and interpretable machine learning
• Emphasis on transparency, reproducibility, and provenance-aware experiments
• Usability, flexibility, and methodological rigor, lowering entry barrier for scientists
• Integrated interpretability tools that produce rules in natural language

## Teaser

Helix is an open-source, FAIR-by-design analytics framework to address the growing need for transparency, interpretability, and reproducibility in data-driven scientific research. Designed for tabular data, Helix integrates the full analytical life cycle within a single modular, extensible environment. Helix places experimental provenance and human interpretability at its core, ensuring that modeling decisions, data transformations, and analytical outcomes are fully traceable and accessible. Its lightweight, browser-based interface lowers technical barriers for domain scientists while preserving methodological rigor and flexibility.

## Introduction

The massive increase in scientific research data generated by modern experimental approaches requires the development and application of robust tools for data analysis and machine learning (ML) that are findable, accessible, interoperable, and reusable (FAIR) as well as interpretable. In domains such as biomaterials science, engineering, chemistry, healthcare, and biosciences, data-driven discovery typically requires interdisciplinary teams. These teams collaborate to implement unbiased data preprocessing strategies, select appropriate modeling techniques, and interpret model outputs to accelerate and inform research outcomes and support rational design and decision-making. This process is often iterative, with experts providing feedback over long periods of time to refine models and optimize the methods employed. In cases where initial analysis identifies issues with the data, such as outliers, unbalanced data classes, or experimental measurement uncertainty, another round of data collection and preprocessing might be necessary. That means that data for the same problem are likely to be analyzed multiple times using different dataset versions and methodological pipelines.

For interdisciplinary co-development of analytics, there is also a need for tools that allow domain experts to focus on interpreting and using analysis results rather than developing code. The widespread use of ML and the overwhelming availability of thousands of community-driven open-source packages in Python and R increase the barrier for interoperable and reusable data analysis methodologies. To facilitate accurate analytics, transparency, and modeling results comparison, there is a strong need for easy-to-use tools that automatically track data, all methodological choices, performance metrics, and modeling results. Recording provenance facilitates rigorous reporting and fosters confidence in the reliability and replicability of outcomes.

Focusing on the FAIR use of ML, Samuel et al. conducted a survey of experts and identified challenges around general ML reproducibility.^1^ Those relevant to provenance include the availability of the source code used and information on the data used for training, testing, and evaluation. Bad practices that should be avoided include a lack of reference implementation, insufficient model parameter description, missing information on software requirements (packages used, package versions, and dependencies, for instance), changes in the code not reflected in the final publication, missing information on modeling methods used, and a lack of documentation on data preprocessing.

While numerous tools exist to support FAIR-aligned analytics and ML workflows for tabular data, they often lack the flexibility or transparency required for rigorous end-to-end team-driven data analysis. Instead, they mostly focus on FAIR data and ML deployment and are often research-domain specific. Hopsworks,^2^ for instance, is an open-source platform that integrates big data versioning, metadata tracking, and feature management, facilitating the deployment of ML pipelines with a focus on FAIR principles. It emphasizes data provenance and reproducibility, ensuring that datasets and models are well-documented and accessible. However, it presents a higher barrier to entry for non-technical users. Its open-source version also limits, for example, the number of models that can be deployed. ProvBook^1^ is a proof-of-concept tool to enhance the reproducibility of ML experiments using Jupyter notebooks. Although it captures the provenance of data and computational steps taken, it assumes that users understand Python. It aims to demonstrate the importance of ML modeling provenance rather than providing an end-to-end tool for analysis. AIF360^3^ and FAT Forensics^4^ target fairness auditing and interpretability of ML models. However, they do not address other aspects of the analytics pipeline, such as data preprocessing. Dalex^5^ is a Python package that provides tools for visualizing and explaining ML models. It supports the interpretability of complex models, making them more transparent, facilitating the understanding and reuse of ML models. SIMON, on the other hand, is an analytics tool that addresses the need for end-to-end analysis and includes data preprocessing, statistical tests, and ML modeling for biomedical data.^6^ It uses a graphical interface that facilitates running the analysis, focusing on supporting results interpretation. It was developed using R but has a few limitations regarding customization and post-training model interpretation. WEKA^7^ is a desktop software containing multiple ML and data analysis algorithms for model training and deployment. WEKA includes tools to preprocess data; train classification, regression, and clustering models; and analyze results. While complete and robust, being widely used for smaller datasets, this tool does not offer provenance recording or interpretation pipelines to complement the predictions of the ML models.

Understanding the rationale behind ML model decisions is critical, particularly in systems where verification, regulatory compliance, ethics, and trust are very important. Post hoc techniques such as feature importance (FI) analysis are widely used to interpret ML outputs by quantifying the contribution of each input variable to the model’s prediction. Accurate FI estimation can support causal inference, domain expert validation, bias identification, and rational design, thereby enhancing model transparency, fairness, and utility. However, the diversity of ML algorithms and FI techniques introduces variability and uncertainty in interpretations. Differences in model structure, learning strategies, and the local/non-linear nature of data can lead to inconsistent FI results. So, there is also a need for tools that are easy to use and can address this uncertainty in model interpretation.

We propose Helix as a suitable framework for addressing the above challenges. It offers an open-source solution that streamlines the experimental analysis process from data input to model interpretation and deployment while keeping a record of the entire analytics decision provenance. Although originally developed for quantitative structure-property relationship (QSPR) modeling in biomaterials discovery, it is broadly applicable to any tabular classification or regression task in many domains. Helix’s functional requirements were driven by the need for a user-friendly, flexible, extensible platform that allows experimentation with different analytics pipelines. It aims to be accessible for experimental specialists with little or no formal data analytics training while also allowing developers to add user-defined models.

## Results

**Figure 1:**
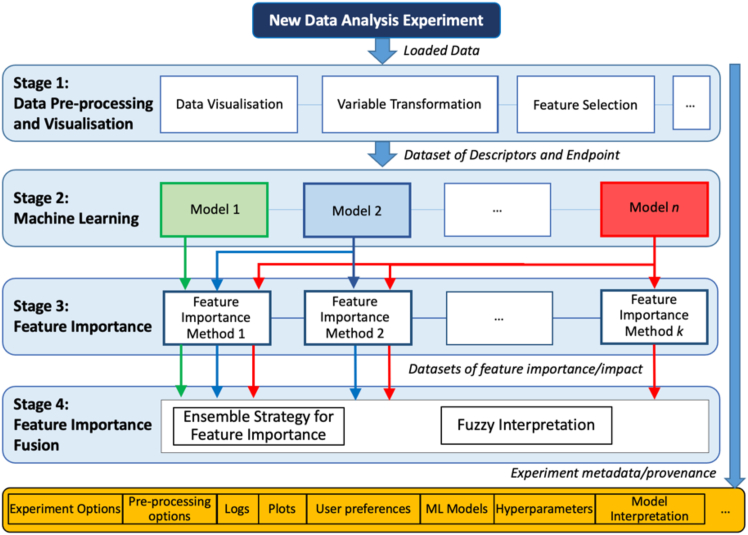
Helix general analytics workflow and provenance recording

Helix was implemented using research software engineering best practices to facilitate modularity, extensibility, and maintainability for developers while keeping a simple graphical interface for non-developer users. It allows for the integration of ML models, preprocessing, and interpretation techniques, facilitating adaptability for future needs. The general analytics workflow of Helix is shown in Figure 1.

Once an experiment is created and data are uploaded, the user can apply several preprocessing and visualization approaches to the data (stage 1 in Figure 1). Helix provides tools for data normalization, transformation, and feature selection. It includes functionalities for generating plots, statistics, correlations, and normality tests, aiding exploratory data analysis. Raw or preprocessed data are then used for ML modeling (stage 2). Users can train and evaluate multiple ML models with options for data training/testing splits and hyperparameter tuning.

Helix implements ensemble FI methods introduced in Rengasamy et al.^8^^,^^9^ (stages 3 and 4, respectively), combining outputs from various models to identify key predictors and linguistic rules that describe the synergistic importance of independent variables in context.

### User interface and additional features

**Figure 2:**
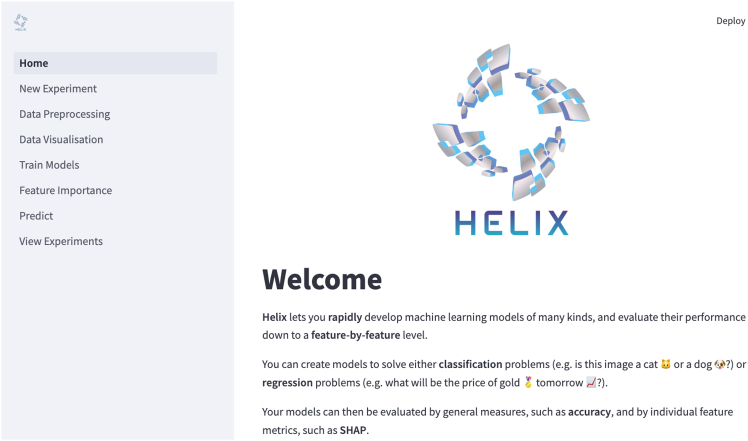
Screenshot of Helix’s welcome page

The graphical user interface (GUI) of Helix is implemented using Streamlit, a library designed for the development of data-centered applications. The decision to adopt Streamlit was due to its operating system agnosticism, lightweight deployment model, minimized development overhead, portability, and native compatibility with browser-based environments. By serving the interface via localhost, Streamlit enables access to Helix directly through the web browser without requiring external servers or very complex installation steps (see Figure 2). This ensures broad accessibility across operating systems (e.g., Windows, MacOS, and Linux) and facilitates adoption.

The interface in Helix is organized into modular components, namely pages, that align with the key stages of the analysis: experiment creation, data preprocessing, visualization, model training, evaluation, ML interpretation, and model deployment. Each module is rendered dynamically, allowing real-time interaction and immediate visual feedback.

### Experiment creation

**Figure 3:**
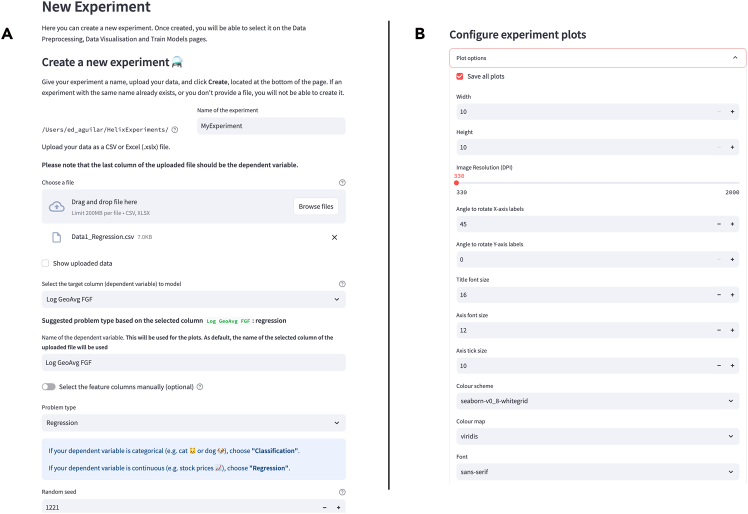
Screenshot of Helix's create experiment page Screenshot of Helix’s create experiment page containing (A) the experiment variables and (B) the plot customization options.

In this module, a user defines general experiment parameters used throughout the pipeline. These include the name of the experiment, the data file, the column containing the target variable (dependent variable), the name of the target variable (used for plotting and logging purposes), the columns containing the independent variables, the problem type (either regression or classification), and a random seed to allow reproducibility of the results (Figure 3A).

### Data preprocessing

Data preprocessing is the first step in Helix’s analytics pipeline. For the independent variables, currently available transformations are standardization (scale each independent variable between −1 and 1 values) and MinMax (scale each independent variable between 0 and 1),^10^ mean centering, mean centering and Poisson scaling, and Pareto scaling. Transformations for the dependent variable in regression problems include natural logarithm, square-root, standardization, and MinMax normalization methods. These can be used to transform the data so they are closer to a normal distribution.

This page also allows the user to apply feature selection to their dataset.^11^ The current methods supported are variance threshold (eliminate features that have a variance below a threshold given by the user), Pearson correlation threshold (variables that have a correlation to another variable greater than a threshold given by the user are eliminated), and least absolute shrinkage and selection operator (LASSO; a sparse feature selection method based on L1 regression).

### Data visualization

**Figure 4:**
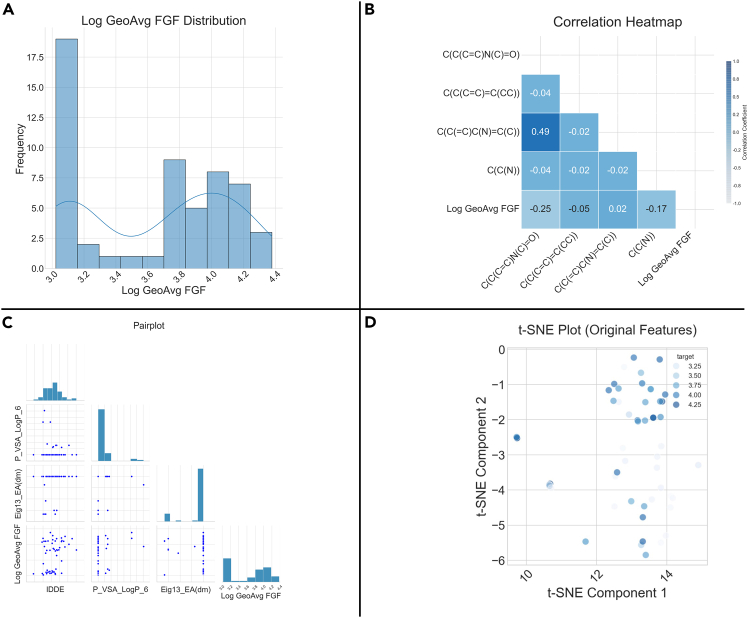
Examples of descriptions available in Helix (A) Distribution plot of the target variable, (B) correlation heatmap, (C) pairplot, and (D) t-distributed stochastic neighbor embedding (t-SNE) plot.

This module provides the user with a series of statistics and graphs for descriptive analytics. Both raw and processed data can be visualized. Examples of graphical descriptions are shown in Figure 4.

### ML modeling

**Figure 5:**
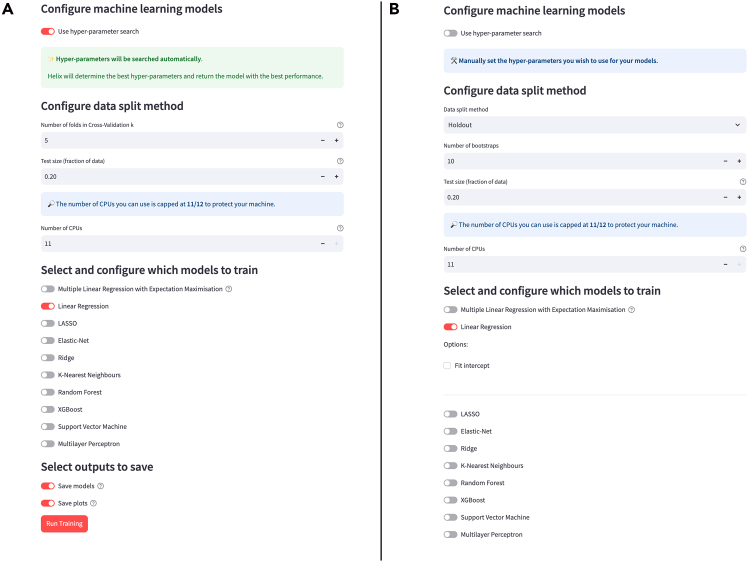
Machine learning model page options (A) Options available when running hyperparameter optimization. (B) Options shown to the user when parameters are selected manually for linear regression.

On this page, the user has different options for modeling, including whether to apply hyperparameter optimization, the number of CPU cores to use for training, how to split the data for the training and test sets, and which ML algorithms to use for training. Currently supported ML algorithms are random forest,^12^ gradient boosting,^13^ support vector machine (SVM),^14^ logistic regression,^15^ Gaussian naive Bayes,^16^ K-nearest neighbors,^17^ and multilayer perceptron^18^ for classification problems. For regression problems, the same algorithms are available, but instead of logistic regression, multiple linear regression,^19^ plus multiple linear regression with expectation maximization (a sparse L1 regression method),^20^ LASSO,^21^ elastic net,^22^ ridge,^23^ K-nearest neighbors,^17^ and multilayer perceptron^18^ are available. To train the models, Helix provides the option to perform hyperparameter optimization via grid search or to set the hyperparameters manually (Figure 5).

**Figure 6:**
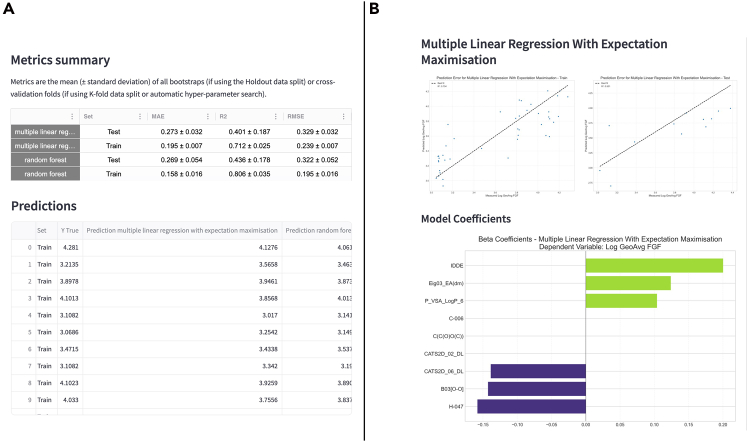
Example of Helix’s output after machine learning model training and evaluation (A) Metrics obtained by the models and the instance-based predictions. (B) Parity plot for each set and the beta coefficients for the linear models.

Once ML training and testing are finalized, results containing performance metrics for each algorithm are shown, along with a table of the predictions of each model for each point (Figure 6A) and plots showing the performance of the models (Figure 6B).

Helix parallelizes the ML model training. To do this, it detects the number of CPU cores that the machine has available and allows the user to select up to all but one core for training (it always leaves at least 1 CPU available to avoid overloading the machine). Then, the ML training process is split among the number of selected CPUs.

**Figure 7:**
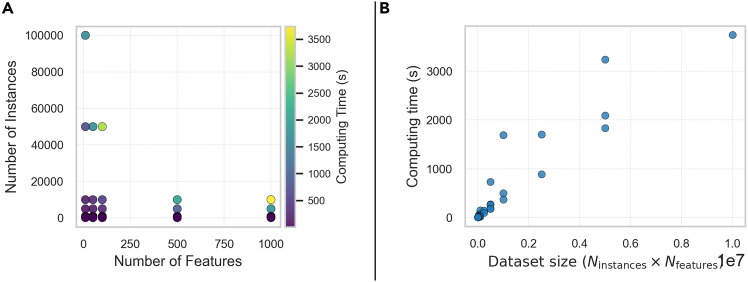
Helix model training times (A) Plot of the number of instances and the number of features colored by the computing time. (B) Plots of computing time as a function of the dataset size.

To evaluate Helix’s performance and computing time, numerous datasets with numbers of instances of 100, 500, 1,000, 5,000, 10,000, 50,000, and 100,000 and with numbers of features of 10, 50, 100, 500, and 1,000 (for the 50,000 dataset, only 10, 50, and 500 instances were used, and for the 100,000 dataset, only 10 features were used) were put into the training pipeline. Using hyperparameter optimization, all available learning algorithms were trained to model these datasets, and the total time was recorded to assess the relationship between computing time and dataset size. This was done using a MacBook Pro M2 Pro and utilizing 12 CPU cores. The result is shown in Figure 7.

We modeled datasets of sizes up to 1e06 under 10 min. For the biggest dataset (10,000 instances and 1,000 features), the total time was 1 h and 2 min. This demonstrates that Helix can model big datasets within a reasonable amount of time. However, this is an extreme case, as the dataset size can be drastically decreased by making use of the feature selection options. Also, because Helix allows the use of multiple cores, the computing times are expected to decrease when using a computer with a greater number of CPU cores, which can allow the analysis of even bigger datasets in faster times.

### Model interpretation

**Figure 8:**
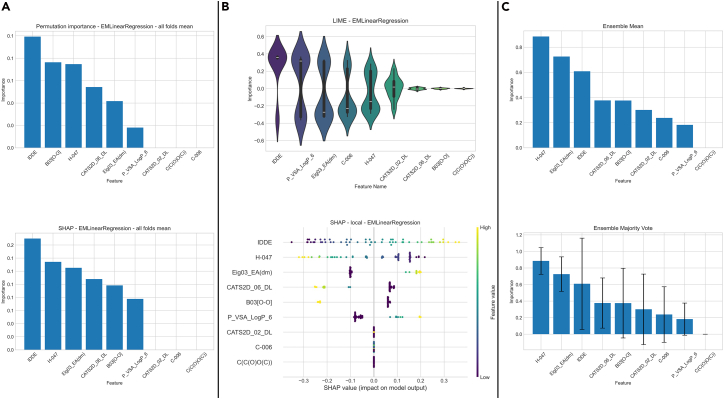
Interpretation results outputs (A) Plots for global feature importance. (B) Plots for local feature importance. (C) Ensemble feature importance plots.

This component of Helix provides insight into which variables most strongly influence a model’s predictions and how changes in individual feature values affect specific outcomes. To support this, Helix implements both global and local model interpretation techniques. Global FI methods, such as permutation importance and SHAP (Shapley additive explanations), quantify the overall contribution of each variable to the model’s predictive performance across the entire dataset. In intuitive terms, these methods indicate which inputs the model relies on most when making decisions on average. Local interpretation methods, including LIME (local interpretable model-agnostic explanations) and local SHAP, focus on individual predictions by approximating the model’s behavior in the neighborhood of a single data point, thereby explaining why a specific prediction was made. In addition, Helix enables ensemble FI analysis, which aggregates importance estimates across multiple models and methods into a single, stable score (e.g., via averaging), reducing method-specific variability and supporting more robust interpretation. Figure 8 shows examples of interpretation results visualization in Helix.

The fuzzy FI fusion uses a fuzzy-logic-driven procedure adapted from Rengasamy et al.^8^ to interpret and contextualize local FI. It selects key features based on user choices or their crisp importance ranks, transforms them into fuzzy categories, computes local importance scores, extracts fuzzy rules, and finally identifies recurring patterns, linking these fuzzy sets to target outcomes and explaining them using natural language if-then rules. This allows data and experimental scientists to understand feature synergy and importance in a more nuanced, human-readable manner, particularly when dealing with complex, non-linear models.

### Model deployment

**Figure 9:**
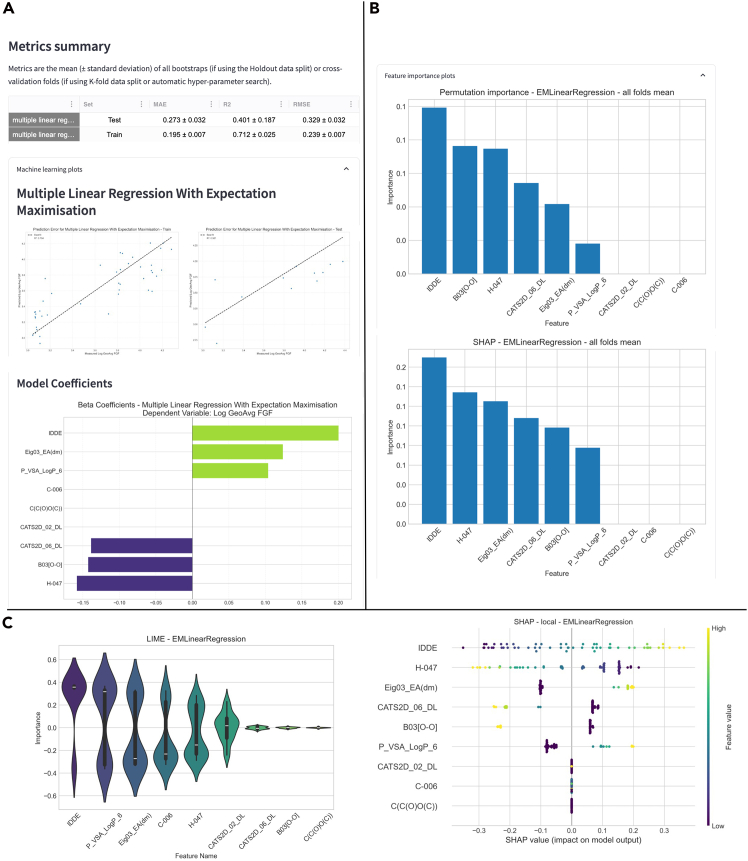
Experiment visualization outputs (A) Machine learning, (B) global feature importance, and (C) local feature importance results.

The predict page allows the user to use the trained models to predict the target variable for their data. For this, the user must provide a CSV file containing all the independent variables that they provided in the original training data. The user will be given the option to select which models are generating new predictions (see Figure 9A). For this, Helix applies the same transformations as those from the original data and provides predictions for each model.

### Experiment inspection and automatic tracking of analysis provenance

Helix offers a page where all analytics results can be visualized (Figure 9). This feature was created to facilitate team communication and analytics workflow auditing, as all results are summarized together with all parameters and options selected in previous pages.

**Figure 10:**
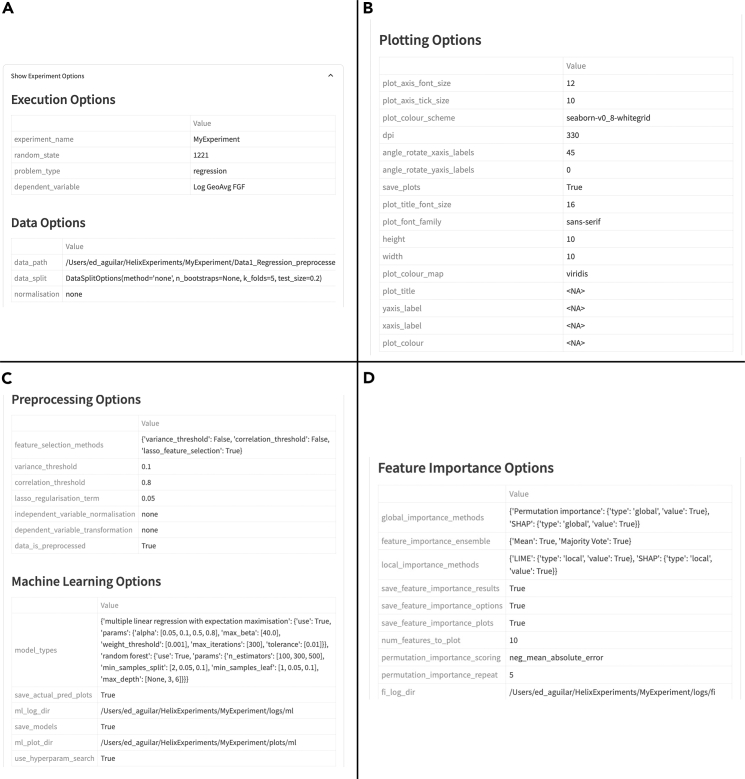
User preferences and parameters stored in Helix interface for all analytics stages (A) Execution and data options. (B) Plotting choices. (C) Preprocessing and machine learning selected and hyperparameters. (D) Feature importance.

**Figure 11:**
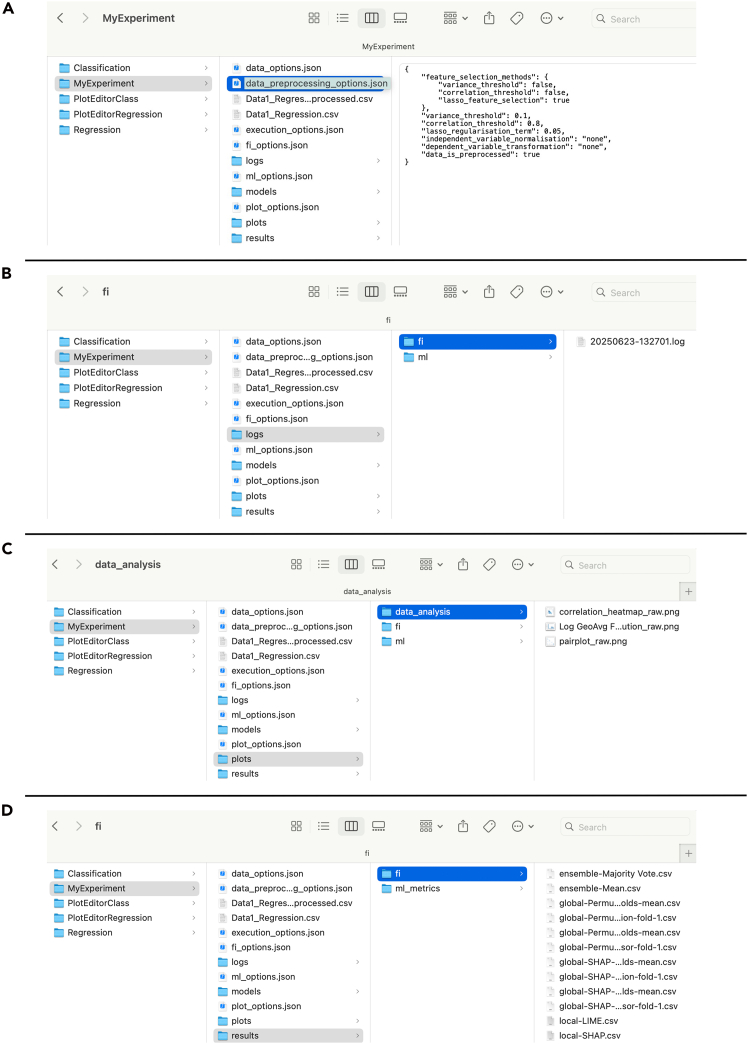
Experiment directory structure (A) Parent directory with all logs and results. (B) Example of a log file for machine learning experiments. (C) Example of plots for the data analysis subdirectory. (D) Example of file structure for feature importance.

For provenance and workflow metadata standardization, the entire analytics experiment, including raw and preprocessed data, preprocessing choices, models, parameters, graphs, and metrics, is saved in a local folder and can be further inspected via the user interface (as shown in Figures 9 and 10) or by browsing a local directory file (Figure 11). The experiment folder can be shared and loaded on any machine that has Helix installed. Log files containing all events that occurred while running the tool, including user choices, analysis workflow steps, and exceptions, are also saved to support developers.

### Use case 1: Biomaterials

Results of analyses to identify microtopographical properties affecting biofilm formation using Helix have recently been published by Romero et al.^24^ In their work, Romero et al. investigated how 2,176 topographical biomaterial shapes embossed into polymers affected colonization and biofilm formation by two types of bacteria, *P. aeruginosa* and *S. aureus*. Using Helix to perform data processing and ML modeling and interpretation modules, the authors were able to successfully create predictive models and extract design rules for topographies influencing biofilm resistance for both cases.

### Use case 2: Cheminformatics

**Figure 12:**
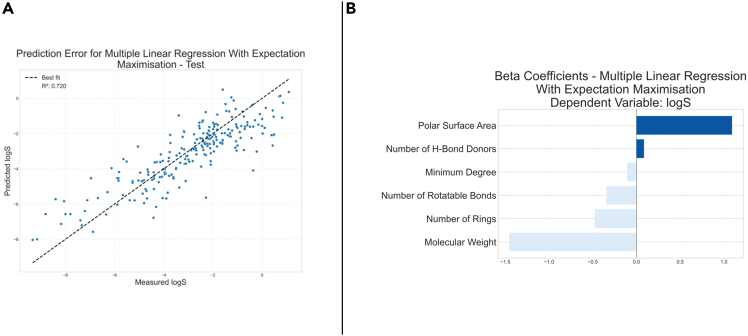
Results of modeling the Delaney solubility dataset with multiple linear regression with expectation maximization using Helix (A) Parity plot of predicted and real logS values. (B) Coefficients of the multiple linear regression.

To demonstrate Helix’s capabilities in chemical sciences, we investigated the Delaney solubility database.^25^ This database comprises the solubility of 1,144 organic compounds, expressed as the logarithm of the solubility in mol per liter (logS). We used the molecule’s minimum degree, molecular weight, number of hydrogen bond donors, number of rings, number of rotatable bonds, and polar surface area as independent variables. The dataset with the descriptors and the target variable was sourced from the DeepChem repository.^26^ No preprocessing of the data was performed for this dataset in Helix. The results of modeling this dataset using linear regression with expectation maximization are shown in Figure 12.

Helix delivered similar results to those reported previously (R^2^ of 0.72 in our case, compared to 0.75 reported by Delaney for the test set).^25^ By analyzing the coefficients of the linear regression, it is possible to understand the impact of each independent variable on the prediction of solubility of the compounds and, from them, either compare to the ground truth or get knowledge of interrelated variables. The coefficients determined match chemical knowledge (higher polar surface area, higher solubility, higher molecular weight, and lower solubility). Although in solubility the interpretation leads to a chemistry concept that is well known, for problems where the relationship between variables is unknown, Helix could help with new hypothesis formulations.

We modeled the same dataset using WEKA and the same ML settings to evaluate the robustness and reproducibility of our tool. WEKA’s modeling yielded an R^2^ of 0.75. The similarity of the metrics of WEKA and Helix demonstrates that our tool is useful to deliver reliable modeling results comparable to established ML tools. However, an advantage of Helix is that it allows further analysis of results, including full customization of plots and FI pipelines that are not provided in most available ML tools. This advantage is critical, as interpretation pipelines can be crucial for understanding which variables impact the model’s prediction the most.

### Use case 3: Medicine

Helix was used in a real-world dataset provided by the Wellcome Leap *In Utero* SWIRL project.^27^ This dataset includes clinical variables collected during pregnancy and aims to predict the risk of fetal demise (stillbirth), making it a high-stakes and sensitive classification task. It consists of 46 samples, each with 90 clinical features. It is highly imbalanced, containing 11 positive cases (classified as near misses) and 35 negative cases (healthy fetal outcomes). To mitigate the risk of overfitting due to the high number of variables relative to the sample size, Helix was used to conduct a two-stage modeling pipeline, with both stages incorporating min-max scaling to normalize feature values to the [0, 1] range.

#### Stage 1: Feature selection

**Figure 13:**
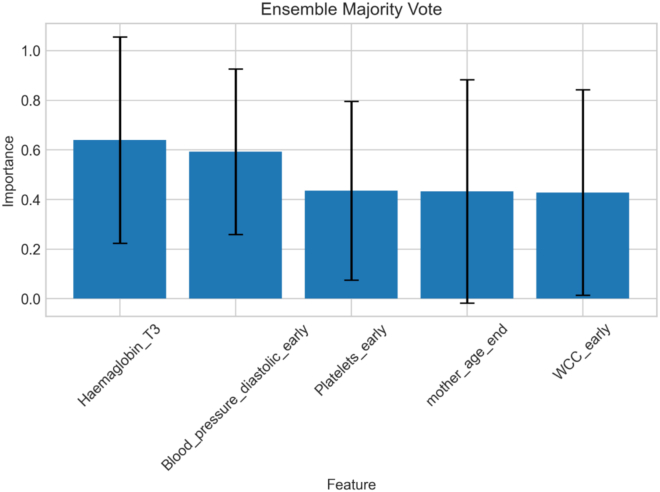
Top 5 features identified from the SWIRL dataset based on a majority vote ensemble of feature importances from 5-fold cross-validation across four classifiers

In the first stage, we employed all four classification models currently supported by Helix—logistic regression, random forest, XGBoost, and SVM—to perform 5-fold cross-validation on the complete dataset. FI was then extracted using Helix’s internal methods, and the results from all models were ensembled using majority voting to select the top five most important features (Figure 13).

The selected features and their clinical descriptions are summarized in Table 1.

**Table 1:** Description of the top 5 selected features from the SWIRL dataset

Feature name	Description
Haemaglobin_T3	hemoglobin measurement in pregnancy at around 28 weeks’ gestation (g/dL)
Blood_pressure_diastolic_early	first diastolic blood pressure measurement taken in pregnancy
Platelets_early	first platelet count (per × 10^9^/L)
mother_age_end	mother’s age at pregnancy end in complete years
WCC_early	first white cell count (per × 10^9^/L)

#### Stage 2: Classification

**Figure 14:**
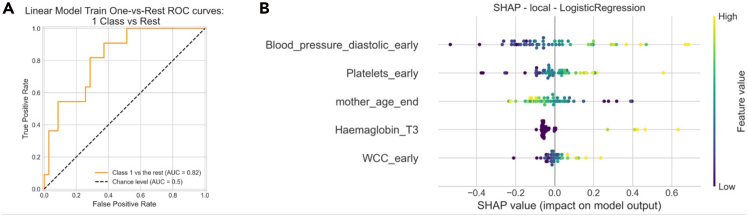
Results of the second-stage analysis on the SWIRL dataset using logistic regression with the top 5 features (A) ROC curve and (B) SHAP plot illustrating the impact of each feature on near-miss predictions.

In the second stage, a logistic regression model was trained using the top 5 selected features. We again applied 5-fold cross-validation on the full dataset. This yielded an accuracy of 0.73 ± 0.06 and an area under the curve (AUC) of 0.73 ± 0.18, where the values represent the mean ± standard deviation across the 5 folds. The performance metrics and model interpretability are illustrated in Figure 14, which includes both the receiver operating characteristic (ROC) curve and the SHAP summary plot.

The SHAP plot reveals that higher diastolic blood pressure, platelet count, and white cell counts in early pregnancy and higher hemoglobin levels around 28 weeks are all positively associated with near-miss cases. Interestingly, the model also suggests that younger maternal age may increase the likelihood of a near miss, a pattern that was later partially validated through expert review. However, experts also noted that this trend could be a result of sample bias due to the limited data size.

To evaluate the robustness and reproducibility of these findings, we further replicated the analysis using WEKA. Since WEKA does not provide a built-in majority voting feature selection method, we replicated stage 1 by applying the same models with similar parameter settings in WEKA and conducting a manual majority vote. The selected features were identical to those obtained using Helix. Furthermore, we repeated the stage 2 experiment in WEKA using the same 5-fold splits, resulting in an accuracy of 0.71 ± 0.14 and an AUC of 0.70 ± 0.13, demonstrating a very similar predictive performance.

To further assess consistency between implementations, we conducted Wilcoxon statistical testing and Pearson correlation and Cohen’s kappa analyses on the predicted labels. The results showed a strong and statistically significant correlation (Pearson r = 0.74, *p* < 0.001) and substantial agreement in classification decisions (Cohen’s κ = 0.70), confirming that the two models produce highly consistent and reproducible categorical predictions. The ROC curves for both software in Figure 13A further support this consistency, demonstrating highly similar discrimination performance between the two implementations. Compared with WEKA, Helix offers a more automated and flexible workflow. Being developed in Python, it can be readily extended to incorporate modern deep learning models such as PyTorch-based Kolmogorov-Arnold networks (KANs), and it automatically generates key visual outputs (e.g., ROC curves, FI plots, and SHAP summaries) that facilitate expert interpretation and communication.

Overall, this case study highlights how Helix can aid experts in uncovering actionable patterns in clinical data, streamline feature selection, and reduce the complexity of medical data analysis while providing a more automated and extensible workflow compared with traditional toolchains such as WEKA. More importantly, Helix was able to expedite the analysis to inform further actions within the research team, including providing proof-of-concept validation of the initial hypothesis around variable importance and how additional data collection should take place.

### FAIR principles and open science

In alignment with the FAIR principles,^28^^,^^29^ Helix’s source code is publicly available on GitHub, with comprehensive documentation hosted online. The software is distributed under the MIT license, encouraging reuse and modification. Installation is facilitated via PyPI, and the platform supports integration with other Python-based tools, enhancing interoperability. Helix also adopts FAIR principles by systematically capturing and storing the provenance of data analytics workflows. By logging detailed metadata at each stage of the ML pipeline—including preprocessing decisions, model configurations, performance metrics, and FI outcomes—Helix ensures that analytical processes are transparent, traceable, and reproducible. This provenance information enables researchers to revisit and audit prior analyses and facilitates knowledge transfer and reuse across team members, projects, and institutions. The structured recording of analytical choices supports interoperability by enabling the integration of Helix outputs into broader computational ecosystems.

### Limitations and future plans

The principal limitation of Helix at present concerns the degradation in computational performance as the dataset size and dimensionality increase. Although Streamlit supports file uploads of up to 200 MB, Helix incurs substantial computational overhead when executing hyperparameter optimization, model training, and interpretability pipelines on large-scale datasets. While these pipelines remain functionally viable and ultimately yield results, the current implementation is optimally suited for datasets comprising up to approximately 5,000,000 cells (defined as the product of the number of features and the number of instances), for which end-to-end execution requires approximately 30 min. To mitigate these scalability constraints, future development will prioritize the adoption of big data technologies, such as Apache Spark, aimed at improving computational efficiency and reducing latency.

The adoption of Streamlit as the GUI framework significantly lowers the barrier to entry for Helix by enabling rapid development, cross-platform compatibility, and an intuitive, browser-based user experience. However, this design choice also introduces important limitations that constrain scalability and collaborative use. Streamlit is primarily optimized for lightweight, single-user applications and does not natively support fine-grained multi-user access control, role-based permissions, or robust authentication mechanisms. In addition, its handling of concurrent sessions and shared states is limited, which can lead to performance bottlenecks and inconsistent behavior in multi-user or long-running analytical workflows. These constraints restrict the suitability of Helix for enterprise-scale deployments or large research teams requiring simultaneous access, persistent sessions, and shared computational resources. Furthermore, Streamlit’s execution model poses challenges for efficient big data processing, particularly when integrating distributed or asynchronous computation. Future development is therefore needed to decouple the user interface from the computational backend and improve support for scalable data-processing frameworks to enable more robust, collaborative, and data-intensive use cases.

A further limitation relates to the restricted diversity of ML algorithms currently available for model training. Moreover, Helix does not presently support unsupervised learning tasks, such as clustering. Planned extensions will therefore focus on expanding the portfolio of supported ML methods and enabling the training and evaluation of clustering models, thereby broadening the range of analytical use cases supported by the platform.

In addition, future work will concentrate on enhancing the analytical toolkit available for model evaluation and comparison. At present, Helix does not provide mechanisms for statistically assessing whether observed performance differences between competing models are statistically significant. Subsequent releases will incorporate formal statistical testing procedures, alongside associated visualization and reporting capabilities, to support more rigorous and reproducible comparative analyses. Furthermore, Helix will also incorporate broader dataset support, including a data search functionality.

To ensure transparency, sustainability, and community-driven development, the project roadmap will be made publicly available through an open backlog. This backlog will be actively used to define development priorities, track and address software defects, and guide the implementation of new features. By exposing this process, the project aims to invite external contributors to participate in the evolution of Helix, fostering collaborative development and accelerating methodological and technical improvements.

## Discussion

Helix represents a significant contribution to the suite of tools available for scientific data analysis, offering a reproducible and interpretable framework for ML on tabular data. Unique to Helix is its integrated approach to provenance-aware, end-to-end analysis and interpretation. In contrast to tools that focus on isolated stages of the analytical pipeline, Helix systematically records the full provenance of each experiment, encompassing data preprocessing, model configuration, training, evaluation, and interpretation, thereby enabling reproducibility, auditability, and comparison across analyses. A key differentiator is its interpretability framework, which extends beyond standard FI methods by employing fuzzy-logic-based information fusion to generate human-readable linguistic rules. These natural language if-then statements capture feature interactions and contextual drivers of model decisions, offering an interpretable abstraction of complex, non-linear behavior. Helix has been successfully applied to several domains, including biomaterials science, chemistry, and medicine. Its open-source nature and adherence to FAIR principles add value for researchers across multiple disciplines. Future developments aim to expand its capabilities to allow for more approaches to data preprocessing, modeling, and interpretation. Although the use of Streamlit makes Helix easy to use, it presents limitations. While well-suited for lightweight, single-user applications, it currently lacks robust support for multi-user access control, concurrent sessions, and advanced state management—features that may be required for larger collaborative deployments or enterprise-scale systems. Future development will aim at addressing these shortcomings and improving big data handling.

## Methods

### Helix architecture

Helix data operations are encapsulated in classes that manage structured data manipulation and transformation. The ML implementation has a *Learner* base class, which abstracts essential operations for model training, including support for cross-validation (holdout and K-fold), bootstrap sampling, and model evaluation using standard metrics. This class enables the addition of user-defined models via inheritance and serves as a foundation for hyperparameter optimization. Both classes conform to a unified interface, enabling integration of classification and regression approaches. Interpretability is managed by the *FeatureImportanceEstimator* class, which supports both global and local instance-based FI analyses. This class employs ensemble methods and allows for the addition of alternative interpretability approaches. There is a services layer, which comprises a suite of functional modules for statistical testing, metric computation, model instantiation, and data preprocessing. These services are decoupled from the domain logic and are injected as dependencies, promoting loose coupling and testability. User interface components and modeling options are similarly modularized, with user input forms, logic, and visualization capabilities segregated into specific modules and organized in pages, as described in the results. The architecture also includes utility modules, such as a class for logging events and various plotting and metric utilities that provide cross-cutting support across the application.

## Resource availability

### Lead contact

Requests for further information and resources should be directed to and will be fulfilled by the lead contact, Grazziela Figueredo (g.figueredo@nottingham.ac.uk).

### Materials availability

This study did not generate any new materials.

### Data and code availability

• All original source code has been deposited at GitHub and is publicly available at https://github.com/Biomaterials-for-Medical-Devices-AI/Helix as of the date of publication and is also available as a PyPI package at https://pypi.org/project/helix-ai/PyPI.
• All original code has also been deposited at Zenodo at https://doi.org/10.5281/zenodo.17978780 and is publicly available as of the date of publication.^30^

## Acknowledgments

This work was supported by the Engineering and Physical Sciences Research Council (EPSRC) under the Materials for Medical Devices project EP/X001156/1.

## Author contributions

Conceptualization, G.F.; methodology, G.F. and J.M.M.; software, E.A.-B., D.L., G.F., K.S., J.M.M., R.O., R.L., T.K., and J.M.-W.; formal analysis, E.A.-B. and G.F.; visualization, E.A.-B.; writing – original draft, E.A.-B., R.L., and G.F.; writing – review & editing, E.A.-B., M.R.A., R.L., D.L., D.A.W., and G.F.; funding acquisition, M.R.A. and G.F.; and supervision, M.R.A., D.A.W., and G.F.

## Declaration of interests

The authors declare no competing interests.
